# Synchronization-based computation through networks of coupled oscillators

**DOI:** 10.3389/fncom.2015.00097

**Published:** 2015-08-04

**Authors:** Daniel Malagarriga, Mariano A. García-Vellisca, Alessandro E. P. Villa, Javier M. Buldú, Jordi García-Ojalvo, Antonio J. Pons

**Affiliations:** ^1^Departament de Física i Enginyeria Nuclear, Universitat Politècnica de CatalunyaTerrassa, Spain; ^2^Neuroheuristic Research Group, HEC Lausanne, University of LausanneLausanne, Switzerland; ^3^Laboratory of Biological Networks, Center for Biomedical Technology, Universidad Politécnica de MadridMadrid, Spain; ^4^Complex Systems Group and GISC, Universidad Rey Juan CarlosMadrid, Spain; ^5^Department of Experimental and Health Sciences, Universitat Pompeu Fabra, Barcelona Biomedical Research ParkBarcelona, Spain

**Keywords:** synchronization, neural mass, Chua oscillators, complex networks, information processing, logic gate

## Abstract

The mesoscopic activity of the brain is strongly dynamical, while at the same time exhibits remarkable computational capabilities. In order to examine how these two features coexist, here we show that the patterns of synchronized oscillations displayed by networks of neural mass models, representing cortical columns, can be used as substrates for Boolean-like computations. Our results reveal that the same neural mass network may process different combinations of dynamical inputs as different logical operations or combinations of them. This dynamical feature of the network allows it to process complex inputs in a very sophisticated manner. The results are reproduced experimentally with electronic circuits of coupled Chua oscillators, showing the robustness of this kind of computation to the intrinsic noise and parameter mismatch of the coupled oscillators. We also show that the information-processing capabilities of coupled oscillations go beyond the simple juxtaposition of logic gates.

## 1. Introduction

It has been established that the healthy brain operates in a highly coordinated way that involves different neural oscillations spanning through multiple spatiotemporal scales (Freeman, 1975; Singer, 1993, 1999; Başar et al., 2000; Varela et al., 2001; Ward, 2003; Buzsáki and Draguhn, 2004). Even though these oscillatory rhythms may have different synchronization properties (Kopell et al., 2000), they have been explained as a result of the balance between excitatory and inhibitory neurons in a network (Börgers and Kopell, 2003; Börgers et al., 2005). In turn, this synchronous firing may subserve complex coordinated patterns of spiking activity which may be transmitted in large neural networks with high temporal accuracy over long distances (Abeles, 1991; Rodriguez et al., 1999; Abeles et al., 2004; Asai et al., 2008; Asai and Villa, 2012; Barardi et al., 2014b). It has also been accepted that the oscillatory activity exhibited by brain signals such as local field potentials (LFP), electroencephalograms (EEG) and magnetoencephalograms (MEG), arises from the synchronized activity of large neuronal assemblies. Such collective dynamics throughout the different scales in the brain is likely to determine the functional role of normal and aberrant synchronization mechanisms during adaptive and cognitive processes as well as brain diseases (Del Prete et al., 2004; Iglesias and Villa, 2010; Pons et al., 2010; Villa and Tetko, 2010). Synchronization's role in coordinating and processing information at different spatiotemporal scales has been also stressed (Lachaux et al., 1999; Stam and de Bruin, 2004; Busáki, 2006; Malagarriga et al., 2015). For instance, synchronization-based selectivity of visual response has been studied in the context of monkeys and cats (Castelo-Branco et al., 2000; Womelsdorf et al., 2006) or even in humans (Rodriguez et al., 1999). Besides, synchronization participates in the odor perception (Stopfer et al., 1997; Laurent et al., 2001; Blumhagen et al., 2011) and coherence of stimuli also affects the selective capability of oscillatory networks (Börgers and Kopell, 2008; Börgers et al., 2008). The processing and computation mechanisms based in all this diversity of synchronized elements has also been studied in detail (Engel et al., 2001; Fries, 2009; Maris et al., 2013; Nikolić et al., 2013; Womelsdorf et al., 2014). Thus, the interaction of different synchronized ensembles of neurons (Womelsdorf et al., 2007; Wulff et al., 2009) plays a role in tasks like learning item-context associations (Tort et al., 2009), selective attention (Fries et al., 2001, 2008; Womelsdorf and Fries, 2007; Bosman et al., 2012), or even conscious perception (Melloni et al., 2007; Levy et al., 2013). At the larger spatial scale in the brain, synchronization participates in the control of task-switching (Phillips et al., 2013) and is studied routinely in normal and abnormal EEG and MEG recordings (Stam, 2005).

Even though much progress in the understanding of these synchronization mechanisms has been gained during many years, it is not fully understood yet how these synchronization relations are established with the participation of different scales simultaneously, or how they operate at the same time without interfering with each other (Barardi et al., 2014a). So, for instance, the information processing capacity of the brain operating under multiple scales has been described very often in terms of logic calculus. At the most microscopic level, the idea of logic calculus based on neuronal activities was embedded in the seminal work of McCulloch and Pitts (1990). Neuronal circuitry performing logic operations was physically implemented in cell cultures of *in vitro* models of selected brain areas (Feinerman et al., 2008; Wolf and Geisel, 2008). This approach is mainly based on action potentials and on the connectivity within the network, rather than on a dynamical analysis of the ongoing activity. At the cellular level neurons have revealed that, in addition to behaving as a bistable system, they can be driven into a continuous oscillation by means of selected voltage-dependent inward currents controlled by intracellular calcium concentrations (Contreras and Steriade, 1995; Hughes et al., 2002; Crunelli et al., 2005). Besides, from the microscopic point of view, neurons may coordinate their firing in response to incoming stimuli, opening the way to a neurocomputing paradigm characterized by different synchronized states where the neurons oscillate with equal frequencies and specific phase relationships (Hoppensteadt and Izhikevich, 2000; Zanin et al., 2011). By associating logical states to the dynamics of coupled oscillators, all usual Boolean operations can be implemented and a full computational model can be obtained (Xu et al., 2004). Beyond the cellular level of neuronal oscillators, it was recently demonstrated that circuits of neurons embedded within a large-scale network of cortical cells were able to express logic functions that are dependent on complex spatiotemporal patterns (Vardi et al., 2013; Menon and Sinha, 2014). This type of analysis can also be made at the mesoscale. Large brain circuits are frequently described as networks of nodes associated with neuronal assemblies, evolving at the mesoscopic scale, in such a way that their dynamics can be considered as that of limit-cycle oscillators subjected to weak forcing and coupling. Phase-reduction theory has revealed synchronization to be among the most relevant features that determine the dynamical states of these systems (Pikovsky et al., 2003; Brown et al., 2004). Furthermore, coupled oscillator theory has established the conditions that allow all the nodes, or a subgroup of them, to operate in one of several synchronization regimes, including complete, lag, generalized, and phase synchronization (Boccaletti et al., 2002; Li and Chen, 2004). In fact, recent work shows that in networks of mesoscopic brain oscillators different forms of synchronization might coexist (Malagarriga et al., 2015). This phenomenon enlarges the processing capacity of neural oscillators, and endows the corresponding networks with stability, flexibility and robustness against perturbations (Zanette, 2004).

In this paper we present a combination of a theoretical approach and its experimental implementation that may shed light on possible mechanisms of brain computation based on synchronized oscillations. Specifically, we show that networks of neural mass oscillators may process inputs in a complex Boolean-like manner. So, the combination of all the fluctuating inputs received by each oscillator in the network determines the global network dynamical response. Every combination of inputs received by the nodes produces a synchronization pattern that relates the dynamics of all the nodes of the network. We interpret the different synchronization states (i.e., phase, generalized, lag, or complete synchronization) of two oscillators in the network as Boolean variables that allow to classify the response of inputs onto pairwise logic gates of different nature. By tuning the characteristics of the oscillatory input acting upon the neural masses, the pairwise coordinated activity changes accordingly. Each form of synchronization brings different information in terms of the synchronized motion in phase space, thus providing additional characterization of such incoming stimuli in the form of coordination evolution. We show that several distinct logical operations can be implemented, in this way, by the same type of neural mass network. However, far from suggesting that the processing capacity of the brain is the result of a more or less complex boolean circuitry, we postulate that it results from its very complex collective dynamics in which synchronization may play a very relevant role (Buzsáki and Draguhn, 2004). The capacity of synchronized oscillations to perform Boolean-like operations is demonstrated by a physical implementation in the form of an electronic system consisting of coupled Chua oscillators driven by oscillatory input signals. Finally, we show that the processing possibilities of larger networks composed by this type of systems go beyond the simple juxtaposition of logic gates.

## 2. Materials and methods

In this Section we describe how the networks of neural mass models and Chua oscillators are implemented. We also describe the numerical methods used and how the synchronization relations between the different nodes of the network are analyzed.

### 2.1. Networks of neural mass oscillators

In order to describe theoretically the computation capabilities of a network of cortical columns acting as a cluster of interacting logic gates, we consider a network of neural mass models as described by Jansen et al. (Jansen et al., 1993; Jansen and Rit, 1995). In the Jansen model, all the neurons in each cortical column are classified into three different groups: pyramidal neurons (responsible of the signal measured in the scalp), excitatory interneurons and inhibitory interneurons. The three populations interact with each other through excitatory and inhibitory connections. Specifically, the population of pyramidal neurons receives both excitatory and inhibitory inputs, in the form of a feedback loop, from the interneuron populations. At the same time, the pyramidal population sends an excitatory input to the interneuron populations inside its cortical column in a feed-forward manner. The pyramidal population also receives an excitatory (inhibitory) input from the pyramidal (inhibitory interneurons) neighboring columns that form the network of neural mass oscillators.

The dynamics of each column in the network is described as follows. Each population converts the average pulse density of action potentials arriving to the population from different origins, ∑*p*_*i*_(*t*), into an average excitatory postsynaptic membrane potential, *y*_*e*_(*t*), through the following expression:

(1) $$\begin{array}{l} \frac{d^{2}y_{e}}{dt^{2}}+2a\frac{dy_{e}}{dt}+a^{2}y_{e}=Aa\sum p_{i}\left(t\right). \end{array}$$

A similar expression with different constant terms, *B* and *b*, substituting *A* and *a*, respectively, works for average inhibitory postsynaptic membrane potentials, *y*_*i*_. *A* (*B*) is related with the maximum height of the excitatory (inhibitory) postsynaptic potential, whereas *a* (*b*) represents the inverse of the membrane time constants and the dendritic delays. At the somas of the neurons forming the populations, the net average postsynaptic potential (PSP) input for the considered population, *m*(*t*) = *y*_*e*_(*t*)−*y*_*i*_(*t*), is transformed into an average density of spikes, *p*_*i*_(*t*) = *C*_*i*_*S*[*m*(*t*)], which will become part of the input of other populations. The transfer function *S*[*m*(*t*)] is given by

(2) $$\begin{array}{l} S\left[m\left(t\right)\right]=\frac{2e_{0}}{1+e^{r\left(\nu_{0}-m\left(t\right)\right)}}, \end{array}$$

where *e*_0_ determines the maximum firing rate of the neural population, ν_0_ sets the net PSP for which a 50% firing rate is achieved and *r* is the steepness of this sigmoidal transformation.

The dynamical activity of the three populations in each cortical column *i* of the network follows from (Malagarriga et al., 2014, 2015):

(3) $$\begin{array}{rcl} \frac{d^{2}y_{0}^{i}}{dt^{2}}+2a\frac{dy_{0}^{i}}{dt}+a^{2}y_{0}^{i} & = & AaS\left[y_{1}^{i}-y_{2}^{i}\right], \end{array}$$

(4) $$\frac{d^{2}y_{1}^{i}}{dt^{2}}+2a\frac{dy_{1}^{i}}{dt}+a^{2}y_{1}^{i}\;=Aa\{C_{2}S[C_{1}y_{0}^{i}]$$

(5) $$\begin{array}{l} +\sum_{j=1}^{N}\alpha_{ij}S[y_{1}^{j}-y_{2}^{j}]\\ +\delta^{i}(t)sin(2\pi f^{i}t+\phi^{i})+{\bar{p}}^{i}+\chi_{i}(t)\}, \end{array}$$

(6) $$\begin{array}{rcl} \frac{d^{2}y_{2}^{i}}{dt^{2}}+2b\frac{dy_{2}^{i}}{dt}+b^{2}y_{2}^{i} & = & Bb\left\{C_{4}S\left[C_{2}y_{0}^{i}\right]+\overset{N}{\underset{j=1}{\sum }}\beta_{ij}S\left[C_{3}y_{0}^{j}\right]\right\}, \end{array}$$

where $y_{0}^{i}$ represents the PSP that feeds the interneurons populations, and $y_{1}^{i}$ ($y_{2}^{i}$) represents the excitatory (inhibitory) PSP that feeds the pyramidal population. The intensity of the excitatory (inhibitory) coupling of columns with their neighboring columns is given by α_*ij*_ (β_*ij*_). Moreover, each column may receive a time dependent input *I*^*i*^(*t*) composed by a constant input, ${\bar{p}}^{i}$, and a periodic external stimulus coming from other brain structures or the sensory system. We represent this periodic input as a sinusoidal driving, i.e., δ^*i*^(*t*)*sin*(2π*f*^*i*^*t*+ϕ^*i*^). Besides, each column may receive a random excitatory contribution onto the pyramidal cells, χ_*i*_(*t*), which can be associated with a stochastic process occurring at a cellular level. The contribution of the column *i* to the EEG activity measured in the scalp is given by $y_{1}^{i}-y_{2}^{i}$. Thus, we will analyze the activity of each cortical column considering the evolution of $y^{i}=y_{1}^{i}-y_{2}^{i}$. The model presented here has an extensive repertoire of dynamical states, being able to produce periodic, quasi-periodic or chaotic (Skarda and Freeman, 1987; Spiegler et al., 2011; Malagarriga et al., 2015) behavior. It also exhibits excitatory/inhibitory segregation (Malagarriga et al., 2014, 2015) depending on the choice of α_*ij*_ and β_*ij*_ values in the network. These coupling parameters also fix a whole set of synchronized regimes that may coexist in the network (Malagarriga et al., 2015). By choosing carefully the input protocol in terms of ${\bar{p}}^{i}$, δ^*i*^(*t*)*sin*(2π*f*^*i*^*t*+ϕ^*i*^), χ^*i*^(*t*), α_*ij*_, and β_*ij*_, we will establish different synchronization-based logic gates.

### 2.2. Networks of chua oscillators

**Figure 1:**
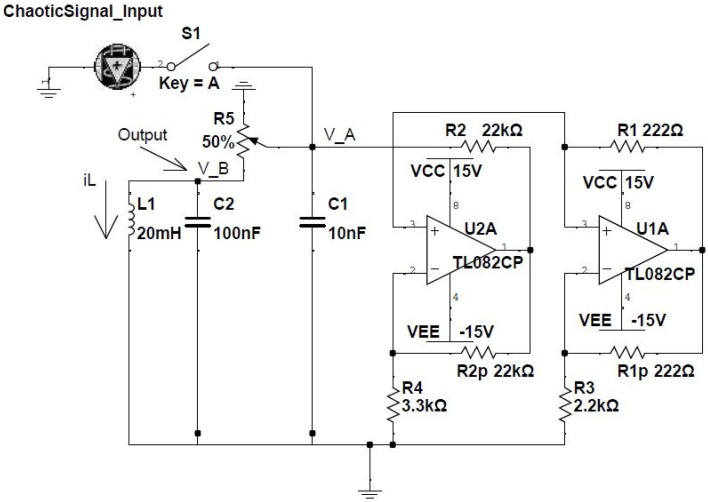
**Electronic implementation of the Chua circuit**. Two TL082 operational amplifiers are the core of the non-linear part of the circuit which follows the function given in Equation 10. The input signal (0/1) is introduced through the capacitor *C*_1_, while the output of the circuit is the voltage (*v*_*B*_) of both *C*_2_ and *L*_1_.

The feasibility and robustness of our theoretical results are proven experimentally by considering a network of electronic oscillators. In this case, the dynamics for every node *i* in the network is determined by the classical Chua circuit (Madan, 1993) (see Figure 1). This system is described by the following equations (Kennedy, 1992; Wagemakers et al., 2007):

(7) $$\begin{array}{rcl} \frac{{dv_{A}}^{i}}{dt} & = & \frac{1}{C_{1}}\left(\frac{{v_{B}}^{i}-{v_{A}}^{i}}{R_{5}}-f\left({v_{A}}^{i}\right)+\frac{{v_{A}}^{i}-s\left(t\right)}{R_{in}}\right), \end{array}$$

(8) $$\begin{array}{rcl} \frac{{dv_{B}}^{i}}{dt} & = & \frac{1}{C_{2}}\left(\frac{{v_{B}}^{i}-{v_{A}}^{i}}{R_{5}}+i_{L}+\frac{{v_{B}}^{i}-{v_{B}}^{j}}{R_{c}}\right), \end{array}$$

(9) $$\begin{array}{rcl} \frac{d{i_{L}}^{i}}{dt} & = & -\frac{{v_{B}}^{i}}{L_{1}}, \end{array}$$

where ${v_{A}}^{i}$ and ${v_{B}}^{i}$ are the voltages of the two capacitors (*C*_1_ and *C*_2_) and ${i_{L}}^{i}$ is the intensity through the coil of the circuit (*L*_1_). *s*(*t*) is an external oscillatory input signal that we can (“1”) or can not (“0”) activate, controlling the dynamical state at which the Chua operates. The strength of the input signal is proportional to the inverse of *R*_*in*_. We will tune its value depending the type of logic gate we will be considering. $f\left({v_{A}}^{i}\right)$ is a piece-wise (non-linear) function given by:

(10) $$\begin{array}{rcl} f\left({v_{A}}^{i}\right) & = & G_{1}{v_{A}}^{i}+\frac{1}{2}\left(G_{1}-G_{0}\right)\left[|{v_{A}}^{i}+B_{p}|-|{v_{A}}^{i}|-B_{p}\right],\; \end{array}$$

with *B*_*p*_ = 1.7 V being the breaking point of the piece-wise function. The values of *G*_1_ and *G*_2_ are obtained from different resistances of the electronic circuit:

(11) $$\begin{array}{rcl} G_{1} & = & \frac{-R_{1}}{R_{1}+R_{3}}+\frac{1}{R_{2}}, \end{array}$$

(12) $$\begin{array}{rcl} G_{0} & = & \frac{-R_{1}}{R_{1}+R_{3}}-\frac{R_{2}}{R_{2}R_{4}}, \end{array}$$

and the rest of the parameters are *V*_*cc*_ = 15V, *V*_*EE*_ = −15V, *R*_1_ = 222 Ω, *R*_2_ = 22 kΩ, *R*_3_ = 2.2 kΩ, *R*_4_ = 3.3 kΩ, *C*_1_ = 10 nF, *C*_2_ = 100 nF, *L*_1_ = 20 mH, and *R*_5_ = 1.38 kΩ.

The coupling circuit between the different Chua circuits (not shown in Figure 1), represented as the last term in Equation (chua_Secondeq), consists on a voltage follower placed at the output of $v_{B}^{j}$ combined with a coupling resistance *R*_*c*_, whose value controls the amount of unidirectional coupling from $v_{B}^{j}$ to $v_{B}^{i}$. The coupling from unit *i* to unit *j* is introduced in a similar way, thus leading to an effective bidirectional coupling. Values of the coupling and input resistances of the different gates implemented in the experiments are summarized in Table 1.

**Table 1:** **Coupling ***R***_***c***_ and input ***R***_***int***_ resistances of the Chua circuits**.

**Node**	***R*_*in*_ [kΩ]**	***R*_*c*_ [kΩ]**	***R*_*int*_ [kΩ]**
**XNOR GATE**			
*A*_1_	0.6 (s(t) → *A*_1_)	3.13 (*A*_1_ → *A*_2_)	100 (*A*_1_ ↔*C*)
*A*_2_	1.2 (s(t) → *A*_2_)	100 (*A*_2_ → *A*_1_)	100 (*A*_2_ ↔*C*)
**AND GATE**			
*B*_1_	32 (s(t) → *B*_1_)	7.22 (*B*_1_ → *B*_2_)	100 (*B*_1_ ↔*C*)
*B*_2_	61 (s(t) → *B*_2_)	96 (*B*_2_ → *B*_1_)	100 (*B*_2_ ↔*C*).

*Note that the coupling strength is proportional to the inverse of the resistances. R_in_ accounts for the resistance of the input signal of each circuit. R_c_ is the coupling resistance of the output signal of each unit to its neighbors. R_int_ is the integration resistance that quantifies the amount of coupling from each unit to the integrator. Parenthesis indicate the direction of the coupling between units i and j, which can be either unidirectional (→) or bidirectional (↔). See **Figure 4** for details*.

The input signal *s*(*t*) used as the “1” input for the logic gates is introduced through the variable *v*_*A*_ by means of a voltage follower and a coupling resistance *R*_*in*_. The signal *s*(*t*) was recorded previously from a Chua circuit with the same parameters as the ones used in the experiments. Both the input and the output signals are characterized by a chaotic behavior (see Madan, 1993 for a detailed analysis of the bifurcation diagram of the Chua circuit, and Wagemakers et al., 2007 for an implementation of a similar circuit in the chaotic regime). Finally, outputs of both gates are sent to an *integrator* system (*C*), by means of a bidirectional coupling, adjusted by an integrating resistance *R*_*int*_ (see **Figure 4** for details).

### 2.3. Numerical methods

**Figure 2:**
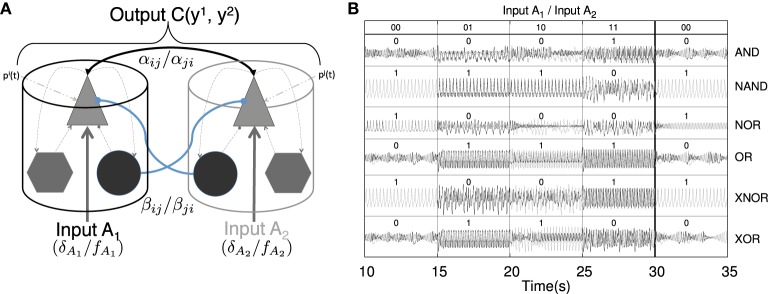
**Implementation of binary logic gates formed by pairwise connected cortical columns**. (**A**) Cartoon depicting two connected cortical columns. Both columns are bidirectionally coupled through both excitatory–excitatory (black solid arrows) and inhibitory–excitatory (blue solid lines) contacts. The intensity of such connections is weighted by α_*ij*_=α_*ji*_ and β_*ij*_=β_*ij*_ coupling strengths, respectively. The pyramidal population within each cortical column additionally receives a constant background pulse density $\bar{p^{i}}$, an oscillatory input defined by an amplitude δ^*i*^ and a frequency *f*^*i*^ and a stochastic input contribution (see Equation 5). The characteristics of these inputs define states which feed this binary logic gate. The out-coming signals, $y^{i}=y_{1}^{i}-y_{2}^{i}$, of the two columns may (*C*(*y*^1^, *y*^2^) ≈ 1) or may not (*C*(*y*^1^, *y*^2^) ≈ 0) be correlated. The correlation value of the timetraces of the two nodes defines the output state of the gate for the given input stimulation (see **Table 3**). (**B**) Several binary logic gates can be obtained from the system shown in panel **(A)** if the appropriate combination of parameters and input protocols are selected (see **Table 2**). Correlated signals, in the form of PS, LS, or CS, result in a “1” state of synchronization, however, not correlated signals are interpreted as a “0” state of synchronization. In the panel **(B)**, both, the time traces of the oscillators and the state resulting from their synchronization are shown for all the binary combinations of input protocols (00, 01, 10, and 11). Several truth tables defined in this way for the logic gates AND, NAND, NOR, OR, XNOR, and XOR are shown.

**Figure 3:**
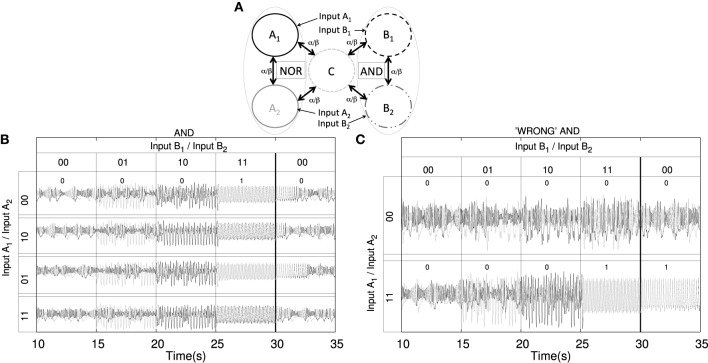
**Implementation of two logic gates embedded in a network of coupled cortical units**. (**A**) Cartoon depicting the network of coupled units. Nodes *A*_1_ and *A*_2_ implement a NOR gate (in a CS regime) while nodes *B*_1_ and *B*_2_ implement an AND gate (in a PS regime). (**B**) The implementation of such logic gates is not altered by the state of synchronization of neighboring pairs, i.e., nodes *B*_1_ and *B*_2_ are capable of implementing a AND gate for any synchronized state of nodes *A*_1_ and *A*_2_ (shown only for a single input state combination for nodes *A*_1_ and *A*_2_). However, the dynamics of *B*_1_ and *B*_2_ are indeed altered, as time traces are no longer the same for each of the four realizations. In fact, such changes in the dynamics may produce long transients before falling into the corresponding synchronized regime (see third pair of time traces between 30 and 35 s). (**C**) In some cases, when tuning the input, the dynamics of the oscillators no longer allows to implement the *desired* logic gates. For an *A*_1_ and *A*_2_ (constant) input configuration of 00, the implementation of an AND gate in nodes *B*_1_ and *B*_2_ fails (see first time traces, where no synchronized output configuration is obtained for the 11 input for *B*_1_ and *B*_2_ as required by the AND truth table). Another type of failure, observed in the second example of timetraces in the panel **(C)**, is the impossibility to return to the initial state of desynchronization. This results in a history-dependent logic gate.

The equations of the Jansen model were numerically solved by means of Heun's method (García-Ojalvo and Sancho, 1999; Toral and Colet, 2014), as performed in Malagarriga et al. (2015). We generated random numbers using standard GSL routines to set different initial conditions and to introduce noise in the dynamics. We implement numerically the noise term χ_*i*_(*t*) using:

(13) $$\begin{array}{l} \chi_{i}\left(t\right)=\sqrt{2\xi_{i}\Delta t}\eta \left(t\right), \end{array}$$

where ξ_*i*_ is the noise amplitude and Δ*t* is the integration time step, whereas η(*t*) is a number resulting from a white noise Gaussian distribution with zero mean and variance equal to 1 (García-Ojalvo and Sancho, 1999). Each simulation of the Jansen model had a time step of 1 ms and ran over a total time of *t* = 25 s in Figures 2, 3, and *t* = 50 s in **Figure 5**. An initial interval of 10 s was omitted to avoid transients in Figures 2, 3, whereas no transient dynamics were found for the simulations presented in **Figure 5**.

### 2.4. Analysis

In order to determine the state of our logic gates, we need to quantify the synchronization between pairs of nodes. Each synchronized state is defined by a characteristic functional relationship between the dynamics of the interacting elements (Boccaletti et al., 2002). Phase synchronization (PS) entails a constant phase difference in time between the coupled oscillators, whereas amplitudes remain uncorrelated (Rosenblum et al., 1996). Generalized synchronization (GS) is characterized by a complex functional relationship between the dynamics of the oscillators that can only be unveiled by the auxiliary system approach (Abarbanel et al., 1996) or the nearest neighbor method (Moskalenko et al., 2012). In turn, lag synchronization (LS) imply a constant time shift between the signals of the two oscillators, with amplitudes being completely correlated, whereas in complete synchronization (CS) no time shift is present. Although CS is not likely to be achieved in a neural context, here we want to emphasize the possibility of the system to reach several synchronized states and, thus, we include here this type of coordinated dynamical evolution as a possible coding state. Accordingly, we computed in the neural mass model the cross-correlation and a phase synchronization index between the output signals of the cortical columns, *y*^*m*^(*t*) (Lachaux et al., 1999). Cross-correlations allow us to distinguish between zero-lag (complete) synchronization (CS) and lag synchronization (LS), whereas the phase synchronization index provides evidence of phase synchronization (PS). Generalized synchronization entails the implementation of a three input logic gate, which has been excluded for the sake of clarity. The phase ϕ^*m*^(*t*) of the output signal is obtained from the Hilbert transform of *y*^*m*^(*t*) (Rosenblum et al., 1996; Mormann et al., 2000). From the phase, the phase synchronization index γ of two oscillators, 1 and 2, is calculated from Δϕ^12^(*t*)=ϕ^1^(*t*)−ϕ^2^(*t*) as (Mormann et al., 2000; Quian Quiroga et al., 2002):

(14) $$\begin{array}{r} \gamma \equiv |{\langle e^{i\Delta \phi^{12}\left(t\right)}\rangle }_{t}|=\sqrt{{\langle \cos\Delta \phi^{12}\left(t\right)\rangle }_{t}^{2}+{\langle \sin\Delta \phi^{12}\left(t\right)\rangle }_{t}^{2}}. \end{array}$$

**Figure 4:**
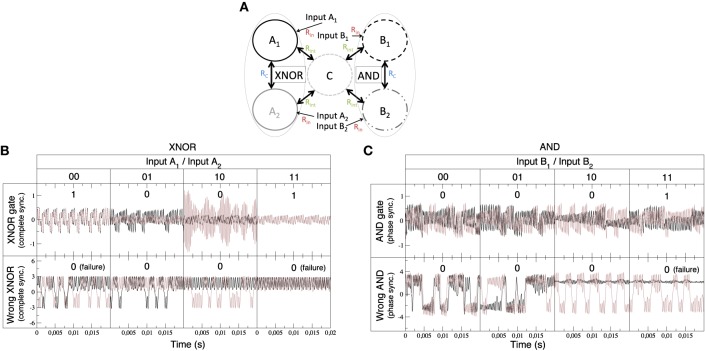
**Experimental implementation of integrated logic gates**. **(A)** Qualitative description of the experimental setup: Nodes *A*_1_ and *A*_2_ form a XNOR gate (in a CS regime) while nodes *B*_1_ and *B*_2_ implement an AND gate (in a PS regime). The output of the two sync-based gates is integrated through node C. **(B)** Time series of the XNOR gate. Functioning of the gate relies on the complete synchronization of units *A*_1_ and *A*_2_. The upper time trace, obtained for low values of the coupling with node C (*R*_*int*_ = 100 kΩ), shows the different outputs of the truth table of the XNOR gate (see **Table 5**). In the bottom signal, coupling with node C is increased (*R*_*int*_ = 25 kΩ), and the gate begins to fail. **(C)** Time series of the AND gate. In this case, the functioning of the gate relies on the phase synchronization of units *B*_1_ and *B*_2_. The upper time trace, obtained for low values of the coupling (*R*_*int*_ = 100 kΩ) with node C, corresponds to the different outputs of the truth table of the AND gate (see **Table 5**). In the bottom time trace, coupling with node C is increased (*R*_*int*_ = 25 kΩ), and the AND gate also begins to fail.

For the Chua circuits, we evaluate the synchronization error as the average of the difference between the outputs of two systems (e.g., units *A*_1_ and *A*_2_ in Figure 4A):

(15) $$\begin{array}{r} \epsilon =\frac{1}{T}\overset{T}{\underset{t=1}{\sum }}|v_{A}^{1}\left(t\right)-v_{A}^{2}\left(t\right)|, \end{array}$$

with *T* being the total number of time steps. We use a similar expression in terms of *y*^1^ and *y*^2^ for the neural mass oscillators. We consider two oscillators to have complete synchronization (or lag synchronization if there is a time shift of the signals) when the values of the synchronization error are lower than a certain threshold ϵ_*th*_ (ϵ_*th*_ = 0.10 V in the case of Chua oscillators, ϵ_*th*_ = 0.01 mV for Jansen oscillators). On the other hand, phase synchronization arises for high values of the phase synchronization index (with a threshold of γ_*th*_ = 0.85 for both cases) and, at the same time, high values of the synchronization error (ϵ > ϵ_*th*_ = 0.10 V, ϵ_*th*_ = 0.01 mV, for the Chua and Jansen oscillators, respectively).

## 3. Results

### 3.1. Small networks of neural mass oscillators

The input received by two Jansen oscillators within a network may determine their state of synchronization (Malagarriga et al., 2015). Thus, labeling the input signals as “0” and “1” (arbitrarily) and the synchronization of the two oscillators as “0” (“1”) when the oscillators are not (are) synchronized, we can interpret the dynamical response of these two nodes to their inputs in terms of binary logic gates. By changing the characteristics of the inputs and the excitatory/inhibitory coupling strengths between the two oscillators, α_*ij*_∕β_*ij*_, several types of binary logic gates may be created.

In order to understand how these clusters of gates operate in networks of oscillators we first study a simpler situation formed only by two bidirectionally coupled Jansen oscillators (See Figure 2, Table 2). In this configuration, each oscillator (*A*_1_ and *A*_2_ from now on) receives an external oscillatory and stochastic input, i.e., Input *A*_1_ (δ_*A*_1__, *f*_*A*_1__, ξ_*A*_1__) and Input *A*_2_ (δ_*A*_2__, *f*_*A*_2__, ξ_*A*_2__), that adds to the other pulse density contributions. The output signal of each oscillator is the difference between the Excitatory Postsynaptic Potential (EPSP) and the Inhibitory Postsynaptic Potential acting upon the pyramidal population ($y^{i}=y_{1}^{i}-y_{2}^{i}$) (see triangles in Figure 2 and the Section 2). The evaluation of the synchronization of the output of the two elements that form the gate, *y*^*i*^ and *y*^*j*^, determines the dynamical response of the system. Other characteristics of the response may also inform about the nature of the inputs received by the oscillators. For instance, it has been shown (Huang et al., 2011; Spiegler et al., 2011) that the driving of Jansen oscillators by a periodic input may result in chaotic, quasi-periodic or periodic dynamical evolutions. So, taken as a whole, both the synchronization state of two oscillators and their dynamical state (e.g., chaotic or oscillatory), inform about the characteristics of the input received by the gate. As mentioned above, we have assigned arbitrarily the “0” and “1” values to the absence of synchronization and its presence, respectively. However, our approach can be generalized in several ways. We could, as well, have labeled each synchronization type (PS, LS, or CS) with a different label increasing the possible number of output states. We could also consider the correlation value to define the output state allowing for fuzzy logic computation. We will not study such complex scenarios, in order to keep our approach as simple as possible.

**Table 2:** **Values of the coupling parameters and input stimulation protocols for inputs labeled as “1” in the implementation of the binary logic gates shown in Figure 2**.

**Parameters**	**Logic gates**					
	**AND**	**NAND**	**NOR**	**OR**	**XNOR**	**XOR**
α_*A*_1_*A*_2__	2.0	27.0	5.0	2.0	27.0	2.0
β_*A*_1_*A*_2__	8.0	5.0	1.0	8.0	5.0	8.0
*p*_*A*_1__(*t*) (Hz)	250	150	155	250	155	250
*p*_*A*_2__(*t*) (Hz)	250	150	155	250	155	250
δ_*A*_1__ (Hz)	120	150	65	250	250	250
δ_*A*_2__ (Hz)	120	150	65	250	250	250
*f*_*A*_1__ (Hz)	10.8	8.5	8.5	8.5	8.5	9.5
*f*_*A*_2__ (Hz)	10.8	8.5	8.5	8.5	8.5	8.5
ξ_*A*_1__ (Hz)	0.0	0.0	1000	0.0	1000	0.0
ξ_*A*_2__ (Hz)	0.0	0.0	1000	0.0	1000	0.0

**Table 3:** **Synchronization errors and phase synchronization indices for each logic gate implemented in Figure 2**.

**Inputs**		**AND**			**NAND**			**NOR**			**OR**			**XNOR**			**XOR**		
***A*_1_**	***A*_2_**	**γ**	**ϵ (mV)**	**Out**	**γ**	**ϵ (mV)**	**Out**	**γ**	**ϵ (mV)**	**Out**	**γ**	**ϵ (mV)**	**Out**	**γ**	**ϵ (mV)**	**Out**	**γ**	**ϵ (mV)**	**Out**
0	0	0.3	0.5	0	0.98	0.001	1	0.99	0.001	1	0.31	0.75	0	0.99	0.001	1	0.19	0.5	0
0	1	0.2	0.7	0	0.97	0.003	1	0.25	0.64	0	0.98	0.42	1	0.23	0.54	0	0.95	0.3	1
1	0	0.1	0.602	0	0.98	0.001	1	0.27	0.73	0	0.97	0.45	1	0.14	0.75	0	0.98	0.45	1
1	1	0.91	0.002	1	0.5	0.3	0	0.63	0.47	0	0.98	0.05	1	0.91	0.2	1	0.24	0.41	0

With all these ingredients we have constructed truth tables based on synchronized states as shown in Figure 2B. The time traces show the online implementation of AND, NAND, NOR, OR, XNOR, and XOR gates that operate in different synchronization regimes. To obtain them, different input protocols and coupling strength relations between the two nodes are needed (see Table 2). However, a single pair of connected oscillators with fixed coupling strengths α_*ij*_∕β_*ij*_ may organize its response in different ways when the input characteristics (frequency, amplitude, etc.) change (e.g., see AND, OR, XOR gates in Table 2). So the same brain circuit represented by a single pair of oscillators may behave as different logic gates depending of the inputs they receive. This ability to classify the response of the system in different ways depending of the inputs received results in a higher flexibility for the information processing capacity of the network. The network is not a static circuit which computes passively the response to the inputs but a whole collection of circuits (based in the logic gates described here) which reconfigures itself depending of the input received. This type of complex networks of logic gates which reconfigure themselves dynamically to adapt its response to specific inputs has been found in other natural systems such as the signaling networks in eukaryotic cells (Domedel-Puig et al., 2011; Rué et al., 2012). As said above, we do not claim, however, that the brain is a circuit of boolean gates but a highly complex dynamical system able to process information in a very sophisticated manner at the mesoscale.

Another trait of these logic gate implementations is related with the complex dynamical evolutions that the neural mass oscillators may show. For instance, for chaotic states, time evolutions depend strongly on the initial conditions. This feature introduces a very strong link between the dynamical evolution of one oscillator and its input. Resulting from this, we argue that our implementation may possess *reversible logic* characteristics (Bennett, 1973), as it allows to recognize from the out coming signals which of the two elements in the logic gate is receiving an input. The details of such paradigm are however out of the scope of this work.

The extension of the previous results to a network of interacting cortical columns leads to spatially distributed computation. In this case, each node in the network receives inputs that determine their synchronization state with other network nodes, following one of the implemented logic gates shown in Figure 2B. In that way, different regions of the network may act as gates that process inputs from the same or different origins, enriching the processing capabilities of the network even further. In this paper, we present a first step toward analyzing these capabilities by considering only relatively simple network motifs. Even if these motifs behave as well-established logic gates in isolation, their behavior when operating within larger networks might be more complex or even unexpected, as shown in Figure 3. In those cases, the whole network can process the whole set of its inputs following complex multidimensional logic rules.

Figure 3A shows a network of five interacting cortical columns that implement two logic gates: NOR (with CS synchronization) and AND (with PS synchronization). The peripheral pairs of columns are capable of working independently, even though their dynamics are influenced by the behavior of the whole network. Figure 3B shows the truth table and the corresponding time traces for the response of the AND gate (columns *B*_1_ and *B*_2_), while columns *A*_1_ and *A*_2_, which implement the NOR gate, receive different inputs. In this case it is worth mentioning that all four time traces for the AND implementation, which are symmetric realizations in terms of initial conditions, display distinctive dynamics depending on the *A*_1_ and *A*_2_ inputs. However, they do not lose the capacity of remaining synchronized/non-synchronized. Nevertheless, some realizations display states of long transient dynamics (see the “0” return state in all pairs of time traces) which are indeed not beneficial for fast brain computation. One possible application of transient dynamics in this system can be related with the ability to discern between the different input scenarios thanks to the different length of the transient dynamical evolutions. Such transient dynamics coding may be related with brain functions as proposed in Rabinovich and Varona (2011). Thus, overall, such dynamical behavior shows that the network implementation of logic gates is stable in terms of synchronization and arises from interdependent dynamics.

Despite being a robust dynamical feature, logic gate implementation strongly depends on the type of input and the coupling strength ranges between columns. In this regard, Figure 3C shows two situations in which there is a wrong output configuration of an AND gate depending on the input applied to nodes *A*_1_ and *A*_2_ (here δ_*A*_1__ = δ_*A*_2__ = 300 Hz, while all other input characteristics are the same as those in Table 4). *B*_1_ and *B*_2_ both receive the standard oscillatory input protocol. In the first pair of time traces shown, the output displayed by nodes *B*_1_ and *B*_2_ does not show correlated dynamics (when *B*_1_ and *B*_2_ receive “1” inputs) as expected for a correct AND implementation. In turn, the second pair of time traces shows how, after displaying a correct output for the initial four pairs of input states, the subsequent return to the first output configuration is no longer possible. Such history dependent behavior entails the impossibility, for these conditions, of a forward implementation of the previous logic gate (AND) but results in the implementation of a state- (or history-) dependent one. These situations result only for specific input patterns. However, the response to other inputs may produce well-behaved logic gates.

**Table 4:** **Values of the parameters used in the implementation of the NOR and AND logic gates appearing in Figure 3**.

**Parameters**	**NOR**	**AND**
**NOR and AND gates**		
α_*ij*_	5.0	2.0
β_*ij*_	1.0	8.0
α_*iC*_ = α_*jC*_	0.01	2.0
β_*iC*_ = β_*jC*_	0.0	1.7
*p*_*i*_(*t*) = *p*_*j*_(*t*) (Hz)	155	250
*p*_*C*_(*t*) (Hz)	250	250
δ_*i*_ = δ_*j*_ (Hz)	65	191
*f*_*i*_ = *f*_*j*_ (Hz)	8.5	10.8
ξ_*i*_ = ξ_*j*_ = ξ_*C*_ (Hz)	0.0	0.0

### 3.2. Small networks of chua oscillators

In the previous section we have shown theoretically that information processing in terms of synchronization-based logic gates for small networks of neural mass oscillators is possible. In this section, we show that these results can also be obtained experimentally by means of electronic circuits. Even though we will use another type of system, the Chua oscillator (Madan, 1993), that can show several dynamic regimes, we are going to consider only situations in which the circuits operate in the chaotic regime (see Section 2.2).

Such an experimental implementation will be a proof of the fact that systems as those considered in the previous section are robust against the parameter mismatch of electronic circuits and the intrinsic noise of real systems. Chaotic systems, such as Chua circuits, may display different kinds of synchronization that can be tuned through the coupling strength between the networked elements and the particular dynamics of an input signal (Arenas et al., 2008). Even though the nature of the oscillators is different from those considered theoretically, these systems have the ability to process information in the same way as those described in the previous section. We have designed and implemented experimentally several logic gates as those described in Figure 2B. Nevertheless, in what follows we are going to focus on the description and integration of a XNOR and AND gates in a network, the former based on complete synchronization and the latter on phase synchronization.

Figure 4A shows schematically the system integrating the outputs of a XNOR and AND gate. This circuit represents a network similar to that studied in Figure 3. It consists of an integrated circuit formed by five Chua oscillators, two of them implementing a XNOR gate (nodes *A*_1_ and *A*_2_), other two forming an AND gate (nodes *B*_1_ and *B*_2_) and the fifth Chua circuit (nodes *C*) integrating the output of both gates. The input of each dynamical unit can be either “1” (when a complex signal is injected into the node) and “0” (in the absence of an input signal). In order to assess the emergence of the synchronized time evolutions we have computed the synchronization errors of the two pairs of nodes that implement the logic gates (see Equation 15). Such errors allow us the determine a proper threshold to define each synchronized state, and thus each output state, in the truth tables of the logic gates. Time traces and the corresponding truth table for the XNOR gate are shown in Figure 4B, while synchronization errors determining the gates' outputs are summarized in Table 5.

**Table 5:** **Synchronization errors and truth tables of the XNOR gate (with complete synchronization) and the AND gate (with phase synchronization) embedded in a network of coupled Chua oscillators. The thresholds to establish synchronized states are shown in Materials and Methods section**.

**Inputs**		**XNOR**			**AND**		
***A*_1_**	***A*_2_**	**γ**	**ϵ (V)**	**Out**	**γ**	**ϵ (V)**	**Out**
0	0	0.991	0.085	1	0.734	0.357	0
0	1	0.750	0.641	0	0.760	0.294	0
1	0	0.885	0.449	0	0.692	0.285	0
1	1	0.994	0.039	1	0.920	0.288	1

**Table 6:** **Values of the parameters used in the implementation of the flip-flop operational gate shown in Figure 5**.

**Parameter**	**Value**	**Parameter**	**Value**
**Flip-flop operational gate**			
α_*SA*_1__	80.0	β_*SA*1_	0.0
α_*A*_1_*A*_2__	10.0	β_*A*_1_*A*_2__	5.0
α_*RB*_2__	10.0	β_*RB*_2__	0.0
α_*B*_1_*B*_2__	9.0	β_*B*_1_*B*_2__	9.0
α_*I*_1_*A*_1__	52.0	β_*I*_1_*A*_1__	9.0
α_*I*_1_*A*_2__	52.0	β_*I*_1_*A*_2__	9.0
α_*I*_2_*B*_1__	10.0	β_*I*_2_*B*_1__	2.0
α_*I*_2_*B*_2__	10.0	β_*I*_2_*B*_2__	2.0
α_*A*_2_*I*_2__	52.0	β_*A*_2_*I*_2__	0.0
α_*B*_1_*I*_1__	10.0	β_*B*_1_*I*_1__	0.0
**Parameter**	**Value**		
*p*_*i*_(*t*) = *p*_*j*_(*t*) (Hz)	155		
*p*_*S*_(*t*) = *p*_*R*_(*t*) (Hz)	57.8		
*p*_*I*_1__(*t*) = *p*_*I*_2__(*t*) (Hz)			
*p*_*A*_1__(*t*) (Hz)	150		
*p*_*A*_2__(*t*) (Hz)	150		
*p*_*B*_1__(*t*) (Hz)	250		
*p*_*B*_2__(*t*) (Hz)	250		
δ_*S*_ (Hz)	220		
δ_*R*_ (Hz)	200		
*f*_*S*_ = *f*_*R*_ (Hz)	8.5		
ξ_*S,R*,*A*_*i*_, *B*_*i*_, *I*_*i*__ (Hz)	1000		

The AND gate is based on phase synchronization, and it is obtained by a fine tuning of the input and coupling resistances. In this case, we must reach a high phase synchronization but preventing complete synchronization, the latter leading to unavoidable (i.e., trivial) matching of the phases of the oscillators. To guarantee that we have phase, and not complete, synchronization we checked at the same time the synchronization error ϵ and the phase synchronization index γ (see Equation 14). A combination of a high ϵ and a high γ is the signature of phase synchronization between the two units forming the gate. Figure 4C shows the time series and the corresponding truth table for the AND gate. Table 5 summarizes the values of ϵ and γ that lead to a successful performance of both gates.

Finally, it is worth mentioning that a tuning of the integrator resistances (see Table 1) allows the correct functioning of the ensemble of gates. When the integrator resistances are decreased (i.e., the coupling with the central node *C* is increased) to values close to *R*_*int*_≤25 kΩ the functioning of the gates begins to fail due to interferences between them (see bottom panels of Figures 4B,C). Such feature was also present in the case of the Jansen oscillators, in which increasing the coupling strength between the peripheral cortical columns and the central node led to an unstable implementation of logic gates.

### 3.3. Neural mass implementation of a flip flop circuit

**Figure 5:**
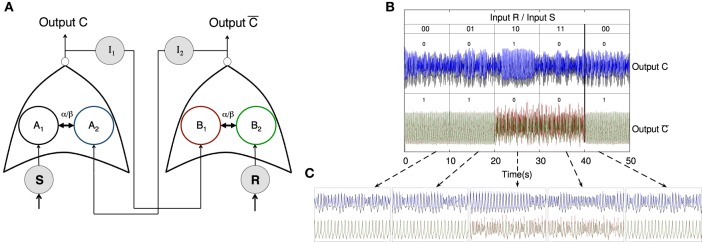
**Implementation of a flip-flop memory**. (**A**) Cartoon depicting the network of cortical columns that fulfill the flip-flop operational gate. *S* and *R* are cortical columns that receive oscillatory inputs, respectively. These columns excite upstream connected columns (*A*_1_ and *B*_2_) which are bidirectionally coupled to columns *A*_2_ and *B*_1_, accordingly. The latter receive inputs from two columns that, in turn, receive the output from *A*_1_ and *A*_2_ (output *C*) and *B*_1_ and *B*_2_ (output $\bar{C}$.) (**B**) Online implementation of a flip-flop. The state of synchronization is preserved when inputs *S* = 0 and *R* = 0, or *S* = 1 and *R* = 0, giving *C* = 0 and $\bar{C}=1$, but “flips” when *S* = 0 and *R* = 1, giving *C* = 1 and $\bar{C}=0$. A not desired situation is the one in which *C* = 0 and $\bar{C}=0$, i.e., neither *A*_1_ and *A*_2_ or *B*_1_ and *B*_2_ are synchronized. This happens when *S* = 1 and *R* = 1, which fulfills a flip-flop truth table. (**C**) Magnification of the time scale of the output signals for the sequence of five *S*−*R* bit pairs of panel **(B)**. The full scale for each sub panel with a pair of signals is corresponding to 5 s.

Other extended systems able to perform more complex logical operations may be implemented by means of networks of coupled oscillators. We have constructed a Set-Reset Flip Flop circuit, which is capable of storing a bit of information at each forward step. To do so, the system has two inputs (Set, *S*, and Reset, *R*) and two outputs *C* and $\bar{C}$, which follow the truth table shown in Figure 5. When neither *S* nor *R* receive an input the output states are *C* = 0 and $\bar{C}=1$. This output state is preserved for the following input step, which is *S* = 0 and *R* = 1. Such feature keeps memory of the previous output state. The following step is *S* = 1 and *R* = 0 which “flips” the output states, being *C* = 1 and $\bar{C}=0$. An undesired state is achieved when both *S* and *R* are 1, which leads to *C* = 0 and $\bar{C}=0$ state. Figure 5A shows the network used to implement such memory: two inputs (*S* and *R*) feed two cortical columns which in turn project unidirectionally toward neural masses *B*_1_ and *A*_2_, via intermediate cortical columns *I*_1_ and *I*_2_, respectively. The state of synchronization of nodes *A*_1_ and *A*_2_ will give the output *C*, whereas the state of synchronization of nodes *B*_1_ and *B*_2_ gives $\bar{C}$. Figure 5B shows the online implementation of the flip-flop memory, with “flipped” output states and an undesired $C=\bar{C}=0$ state in which none of the pairs *A*_1_–*A*_2_ or *B*_1_–*B*_2_ are synchronized. Note that transients affect the performance of the flip-flop implementation (see third pair of time traces below Figure 5B) but such obstacle may be dependent on initial conditions. Moreover, as in previous implementations, each output state is characterized by distinctive time evolutions, which, on top of memory storage, also give information about which terminal (*S* or *R*) receives the input.

## 4. Discussion

In order to address how information can be processed, from the perspective of synchrony, at the mesoscopic scale we have analyzed theoretically a network of coupled neural mass oscillators. This network uses synchronization as the essential ingredient to process the information arriving to/from each of its nodes. We have seen that by interpreting the inputs arriving to the oscillators of the network as “0” or “1” and defining the output of the binary operation in terms of the synchronized state of the two oscillators also as “0” or “1”, several binary logic gates can be constructed. This dependence of the synchronization level of two columns on their stimulation has been observed experimentally, for instance, in the cat visual cortex (Gray et al., 1989). Interestingly, different binary logic gates constructed using the same physiological circuitry result only from changes in the input signals received by the oscillators (e.g., AND, OR, and XOR gates in Figure 2). This rich behavior shown by only two coupled cortical columns may be very fruitful when many other columns are considered. In this sense, the ability to analyze input signals with very different characteristics (average density of spikes, amplitude and frequency of oscillations or noise level) is multiplied by the simple addition of this type of binary logic gates in a network. Nevertheless, this simplistic view may be even more sophisticated when putting the binary motifs together in a larger network. As shown in Figure 3, simply by connecting two different logic gates through a hub may result in a system where the two gates operate in parallel independently of each other (Figure 3A) or operate in a different way (Figure 3B). In this case, outputs may depend on the input of both gates at the same time or on the history of the input states driving the nodes (see examples of both behaviors in Figure 3B). This type of dynamics, in larger networks, makes selectivity of the state in terms of the input protocol even richer than just the repetition of simple binary logic gates in a network. In order to show the generality of this type of networks (and also its robustness in terms of the dynamical oscillators used to build the network) we have constructed several binary logic gates with electronic circuits operating in a chaotic regime. We have shown experimentally that a network built by coupling two of these gates through a hub (using the same simple motif as before) is able to process information as expected (see Figure 4). Finally, we have shown theoretically that by using a network of oscillators we can implement a Set-Reset Flip Flop circuit (Hahnloser et al., 2000), which is an example of another stimulus selector, in this case, that is able to store information.

To conclude, it is worth mentioning that, in this work, we have considered only the simplest interpretation of input and output states (leading to Boolean logic). However, our results may be analyzed in a wider view, for instance, if we explicitly consider the degree of synchronization of the different elements (resulting in fuzzy logic) or if we consider as possible output states all types of synchronization (phase, generalized, lag, complete, …) between the different elements which form the network of oscillators. The fact that we consider only one of the different dynamical characteristics of the system, in our case its degree of synchronization, is a coarse simplification. The dynamical response of the network is not determined only by its degree of synchronization. For instance, the frequencies involved in the dynamics, or the degree of excitation/inhibition segregation, may also inform about the input stimulus characteristics, enlarging in this way the computational capabilities of the system.

### Conflict of interest statement

The authors declare that the research was conducted in the absence of any commercial or financial relationships that could be construed as a potential conflict of interest.
